# Parental and child genetic burden of glycaemic dysregulation and early-life cognitive development: an Asian and European prospective cohort study

**DOI:** 10.1038/s41398-023-02694-x

**Published:** 2024-01-04

**Authors:** Jian Huang, Michelle Z. L. Kee, Evelyn C. Law, Ka Kei Sum, Patricia Pelufo Silveira, Keith M. Godfrey, Lourdes Mary Daniel, Kok Hian Tan, Yap Seng Chong, Shiao-Yng Chan, Johan G. Eriksson, Michael J. Meaney, Jonathan Yinhao Huang

**Affiliations:** 1 Singapore Institute for Clinical Sciences (SICS), Agency for Science, Technology and Research (A*STAR), Singapore, Singapore; 2 Department of Epidemiology and Biostatistics, School of Public Health, Imperial College London, Norfolk Place, London, UK; 3 Department of Paediatrics, Yong Loo Lin School of Medicine, National University of Singapore, Singapore, Singapore; 4 Department of Paediatrics, Khoo Teck Puat-National University Children’s Medical Institute, National University Hospital, Singapore, Singapore; 5 Population Health Sciences, Bristol Medical School, University of Bristol, Bristol, UK; 6 Department of Psychiatry, Faculty of Medicine and Ludmer Centre for Neuroinformatics and Mental Health, Douglas Hospital Research Centre, McGill University, Montreal, Quebec Canada; 7 MRC Lifecourse Epidemiology Centre and NIHR Southampton Biomedical Research Centre, University of Southampton & University Hospital Southampton NHS Foundation Trust, Southampton, UK; 8 Department of Child Development, KK Women’s and Children’s Hospital, Singapore, Singapore; 9 Department of Maternal Fetal Medicine, KK Women’s and Children’s Hospital, Singapore, Singapore; 10 Department of Obstetrics & Gynaecology, National University Health System, Singapore, Singapore; 11 Yong Loo Lin School of Medicine, Human Potential Translational Research Programme, National University of Singapore, Singapore, Singapore; 12 Department of Obstetrics & Gynaecology, Yong Loo Lin School of Medicine, National University of Singapore, Singapore, Singapore; 13 Department of general practice and primary health care, University of Helsinki, Helsinki, Finland; 14 Folkhälsan Research Center, Helsinki, Finland; 15 Brain-Body Initiative, Agency for Science, Technology and Research (A*STAR), Singapore, Singapore; 16 Centre for Quantitative Medicine, Duke-NUS Medical School, Singapore, Singapore; 17 Thompson School of Social Work & Public Health, Office of Public Health Studies, University of Hawai’i at Mānoa, Honolulu, HI USA

**Keywords:** Genomics, Predictive markers, Molecular neuroscience

## Abstract

Insulin resistance and glucose metabolism have been associated with neurodevelopmental disorders. However, in the metabolically more susceptible Asian populations, it is not clear whether the genetic burden of glycaemic dysregulation influences early-life neurodevelopment. In a multi-ethnic Asian prospective cohort study in Singapore (Growing Up in Singapore Towards healthy Outcomes (GUSTO)), we constructed child and parental polygenic risk scores (PRS) for glycaemic dysregulation based on the largest genome-wide association studies of type 2 diabetes and fasting glucose among Asians. We found that child PRS for HOMA-IR was associated with a lower perceptual reasoning score at ~7 years (β = −0. 141, *p*-value = 0.024, 95% CI −0. 264 to −0. 018) and a lower WIAT-III mean score at ~9 years (β = −0.222, *p*-value = 0.001, 95% CI −0.357 to −0.087). This association were consistent in direction among boys and girls. These inverse associations were not influenced by parental PRS and were likely mediated via insulin resistance rather than mediators such as birth weight and childhood body mass index. Higher paternal PRS for HOMA-IR was suggestively associated with lower child perceptual reasoning at ~7 years (β = −0.172, *p*-value = 0.002, 95% CI −0.280 to −0.064). Replication analysis in a European cohort, the Avon Longitudinal Study of Parents and Children (ALSPAC) birth cohort, showed that higher child PRS for fasting glucose was associated with lower verbal IQ score while higher maternal PRS for insulin resistance was associated with lower performance IQ score in their children at ~8.5 years. In summary, our findings suggest that higher child PRS for HOMA-IR was associated with lower cognitive scores in both Asian and European replication cohorts. Differential findings between cohorts may be attributed to genetic and environmental factors. Further investigation of the functions of the genetic structure and ancestry-specific PRS and a more comprehensive investigation of behavioural mediators may help to understand these findings better.

## Introduction

The global burden of diabetes has been increasing in past decades, with Southeast Asia among the regions with the highest burden [1]. A similar trend has been observed for early-onset type 2 diabetes in children, adolescents, and young adults [2]. Recent genome-wide association studies (GWAS) of type 2 diabetes, showed that individuals of East and South Asian ancestry shared a substantial genetic susceptibility similar to those of European ancestry [3, 4]. However, novel genetic loci for type 2 diabetes have also been reported in Asian GWAS, suggesting ancestry-specific susceptibility to insulin resistance and/or secretion may exist [3]. Aside from their obvious metabolic sequelae, insulin resistance in the central nervous system and cerebral glucose metabolism have also been associated with neurodegenerative diseases [5, 6], despite brain glucose uptake being insulin-independent [7]. Childhood metabolic health and type 2 diabetes during adolescence have also been associated with poorer performance in cognitive assessments [8, 9]. However, it is not clear whether genetic predisposition to glycaemic dysregulation plays a role in early-life cognitive function, particularly in the understudied Asian populations with a greater metabolic susceptibility and in whom genetic influences are less well studied [10].

Polygenic risk scores (PRS) can be used to quantify genetic risk for a specific trait or disease [11]. PRS for type 2 diabetes was found to be associated with a higher risk of vascular dementia [12, 13]. Potential underlying mechanisms are unclear but some hypotheses have been proposed, including glycemic variability and brain insulin resistance [14, 15]. However, most GWAS have been conducted among populations of European ancestry, limiting the predictive power of PRS in the less represented ethnic groups due to differences in effect sizes of risk alleles, allele frequencies, and linkage disequilibrium [16, 17]. Novel loci identified in Asian populations further suggest that genetic risk for type 2 diabetes can be more reliably estimated using ancestry-specific GWAS [3, 4]. Recent GWAS of type 2 diabetes and fasting glucose in Asian populations have provided an opportunity to study the degree to which the genetic burden of child glycaemic dysregulation influences early-life neurodevelopment in this less represented population. However, it is not clear whether genotypic associations are reflective of the children’s own genetic risks or the influences of the parental (maternal and paternal) genomes. Parental genomes not only influence offspring’s health via direct effect of the transmitted alleles but also indirect effect, i.e., genetic nurture [18]. Thus, in this study, we constructed PRS for child homoeostatic model assessment for insulin resistance (HOMA-IR) and fasting glucose for the children and their parents in the Growing Up in Singapore Towards healthy Outcomes (GUSTO) birth cohort study and investigated their relationships with mid-childhood neurodevelopmental outcomes (~7 to ~9 years). We also investigated potential mediating mechanisms underlying the effect of genetic burden for child glycaemic dysregulation on cognitive function. To replicate the findings, we examined if associations were similar in a European cohort, the Avon Longitudinal Study of Parents and Children (ALSPAC) birth cohort.

## Materials and methods

### Data source

This study was conducted within a multi-ethnic prospective cohort study consisting of Singaporean children of homogenous parental Chinese, Indian, and Malay ancestries [19]. Between June 2009 and September 2010, the GUSTO study recruited pregnant women aged at least 18 years attending their first-trimester antenatal ultrasound scan at one of Singapore’s two major public maternity units, namely National University Hospital and KK Women’s and Children’s Hospital. The original GUSTO cohort consisted of 1450 pregnancies, but 246 were lost to follow-up before delivery, 96 had conceived by in vitro fertilisation, and 10 pairs of twins were excluded, resulting in a sample of 1095 children for analysis in the present study. Obstetric information was obtained from medical records using standardised forms, and sociodemographic characteristics were collected through self-report questionnaires during pregnancy. Cord tissue (children), maternal blood collected during pregnancy week 26 (mothers), and paternal buccal swab samples collected about two to three years after the birth of the child (fathers) were used to genotype the cohort participants and their parents. The GUSTO cohort is deeply phenotyped during multiple pregnancy and postnatal visits focusing on maternal health, child growth and development, as well as paternal factors. Specifically, fasting glucose (mmol/L) and fasting insulin (mU/L) were measured between 6 to 8 years old. HOMA-IR was calculated as (fasting glucose × fasting insulin)/22.5. Genotyping data were available for 1025 mother-child dyads and 689 fathers after the quality control procedure.

In this study, we focused on cognitive function assessed during the mid-childhood follow-up. Wechsler Abbreviated Scale of Intelligence 2^nd^ Ed (WASI-II) was administered at age ~7 years (*N* = 481) and Wechsler Individual Achievement Test 3^rd^ Ed (WIAT-III) was assessed at age ~9 years (*N* = 366). In GUSTO, two subtests of WASI-II were administered, i.e., block design task and matrix reasoning test. A perceptual reasoning index was derived from the evaluation of these subtests, representing non-verbal fluid intelligence and visuomotor or coordination skills [20]. WIAT-III is a standardised academic achievement test assessing the ability of mathematics, reading, spelling, and oral language [21]. A WIAT-III mean score was calculated based on the standardised tests on the subscales. For both WASI-II perceptual reasoning index and WIAT-III mean score, we further standardised them and used a per standard deviation (SD) unit in the association analyses. WASI-II perceptual reasoning score and WIAT-III mean score were moderately correlated in our cohort (*r* = 0.312).

### Genotyping and imputation processing

Child and parental genotyping for the GUSTO cohort was performed using the Infinium OmniExpressExome array. For each ethnicity, we removed genetic variants with call rates <95%, minor allele frequencies (MAF) < 0.05, and *p*-value for Hardy-Weinberg equilibrium ≤10^–6^. Allele frequencies were compared to those in the 1000 G reference panel (East Asian population for Chinese and Malay; South Asian population for Indian) and genetic variants with an allele frequency differing more than 0.2 for Chinese and Indian or 0.3 for Malay were excluded. The resulting data were pre-phased using SHAPEIT v2.837 with family trio information and then imputed using the Sanger Imputation Service. Imputed data with an INFO score >0.8 were retained. After quality control, genotyping data were available for 1,025 mother-child dyads and 689 fathers.

### Construction of polygenic risk scores (PRS)

We obtained summary statistics for the sex-specific GWAS of type 2 diabetes among East Asians (males: 28,027 cases and 89,312 controls; females: 27,370 cases and 135,055 controls) [3] and a GWAS of fasting glucose among East Asians (*N* = 288,127) [22]. Genetic correlation estimated using linkage disequilibrium Score Regression [23] was 0.69 (*P*-value = 5.2 × 10^–78^) between the two GWAS [3, 22].

Our goal in constructing a PRS is to quantify the polygenic risk for glycaemic dysregulation, specifically for HOMA-IR and fasting glucose. Pre-defining a cut-off for linkage disequilibrium and *p*-value thresholding in the GWAS can result in PRS that are not correlated with the traits of interest in the target cohort. Therefore, we used a clumping and thresholding approach to construct PRS by sex and ethnicity. PRS were calculated as the sum of risk alleles in each individual weighted by the effect size estimate obtained from the GWAS. Specifically, we constructed PRS for HOMA-IR based on the GWAS of type 2 diabetes and PRS for fasting glucose based on the GWAS of fasting glucose. We selected single nucleotide polymorphisms (SNPs) using different combinations of *p*-value thresholds (5 × 10^–8^, 5 × 10^–6^, 5 × 10^–4^, 0.01, 0.05, 0.25, 0.5, 0.75, or 1) and clumping R^2^ (0.001, 0.01, 0.1). For each sex- and ethnicity-specific subgroup, we selected the PRS with the highest Pearson’s correlation with child HOMA-IR and fasting glucose.

### Statistical analysis

In our main analysis, we investigated the associations of standardised child, maternal, and paternal PRS for child HOMA-IR and fasting glucose with cognitive function using linear regression. We adjusted for ethnicity using the first three principal components (PCs) of the child’s genotype. We adjusted for confounders associated with child neurodevelopment and precision variables related to glycaemic trait measurement, including child sex, age at glycaemic trait measurement, age at cognitive assessment, the highest maternal education level attained, monthly household income, maternal age at delivery, parity, maternal pre-pregnancy body mass index (BMI), gestational age of child at birth, and maternal status of gestational diabetes mellitus (GDM) [24]. For analysis of child PRS, we additionally adjusted for maternal and paternal PRS based on the identified child PRS SNPs to account for the effect of parental genomes.

Since sex differences have been reported for both metabolic health and neurodevelopment [25, 26], we performed sex-stratified analyses, planned a priori, for both the linear regression model and instrumental variable analysis. Models were similarly adjusted for as those in the main analysis, except that child sex was not included. Robust estimation of standard errors, *p*-values and 95% confidence intervals (95% CIs) were reported.

#### Multiple testing

We accounted for multiple comparisons of two PRS traits (HOMA-IR and fasting glucose), three samples (children, mothers, and fathers), three sex groups (boys, girls, and overall), and two cognitive assessments using Bonferroni correction with a *p*-value threshold of 0.05/(2 × 3 × 3 × 2) = 0.00139. Associations with a *p*-value < 0.05 were considered suggestive findings.

#### Mendelian randomisation

To investigate if the higher polygenic risk for HOMA-IR or fasting glucose influences cognitive function via their effects on these corresponding glycaemic traits, we performed Mendelian Randomisation, i.e., instrumental variable analyses with child PRS as the instrument using two-stage least-squares (2SLS) estimation. For each analysis, we adjusted for ethnicity using the first three PCs based on child genotype, child sex, age at glycaemic trait measurement, and age at cognitive assessment.

#### Mediation analyses

We further examined other potential mechanisms underlying the effect of polygenic risk for insulin resistance and high fasting glucose on cognitive function. We performed mediation analysis using a regression-based counterfactual effect decomposition that allows for exposure-mediator interactions [27]. Pure and total (including interaction) direct effects and pure and total (including mediated-interaction) indirect effects were estimated. We defined direct effects as the effects of genotype independent of glycaemic traits and indirect effects as the effects of genotype due to their effect on glycaemic traits. Mediation analysis for each potential mediator was performed separately. We focused on the potential mediating effects of maternal factors (pre-pregnancy BMI, gestational weight gain, maternal status of GDM, fasting glucose during pregnancy, blood pressure during pregnancy, Edinburgh Postnatal Depression Scale (EPDS) at pregnancy week 26), paternal BMI (~2 years after the childbirth), foetal growth, gestational age of child at birth, birth weight, duration of breastfeeding, childhood BMI (measured at follow-up visits between 3 to 6-year-old), and childhood C-reactive protein (CRP, measured at 6 year-old). When using child PRS or paternal PRS as the exposure, we included the same covariates as in the main association analyses. When using maternal PRS as the exposure, we excluded pregnancy-related covariates because these could be influenced by maternal PRS and contribute partly to mediating pathways.

#### Sensitivity analysis

Since birth weight and childhood obesity are major risk factors for later type 2 diabetes [28, 29] and have been associated with lower cognitive scores [30, 31], we examined if the SNPs used to construct PRS overlap with genes associated with birth weight and child BMI at a genome-wide significant level (*p*-value < 5 × 10^–8^). Since there are no large Asian studies, we obtained GWAS of birth weight (*N* = 298,142 new-born of European ancestry) and child BMI (*N* = 39,620 children of European ancestry aged 2 to 10 years old) from the Early Growth Genetics Consortium [32, 33]. We annotated the SNPs to the closest genes within ±500 kb from their genomic locations. Additionally, we replicated the main analysis using PRS for glycaemic traits excluding the SNPs annotated to birth weight and child BMI genes.

### Replication analysis

We performed a replication analysis to examine if similar associations exist among individuals of European ancestry using the Avon Longitudinal Study of Parents and Children (ALSPAC) birth cohort [34, 35]. Pregnant women resident in Avon, UK with expected dates of delivery between 1st April 1991 and 31st December 1992 were invited to take part in the study. The initial number of pregnancies enroled was 14,541 and 13,988 children were alive at 1 year of age. The total sample size for analyses using any data collected after the age of seven is therefore 15,447 pregnancies and 14,901 children alive at 1 year of age.

ALSPAC mothers and children were genotyped using the Illumina human660W quad and the Illumina HumanHap550 quad array, respectively. The raw genome-wide data were subjected to standard quality control methods as described in the ALSPAC OMICs Data Catalogue [36]. In this study, we included 11,376 unrelated participants of White Caucasian ethnicity, among which genotype data were available for 6923 children and 6623 mothers. PRS for HOMA-IR and fasting glucose were constructed using the same clumping and thresholding method described above for the GUSTO cohort. PRS for HOMA-IR and fasting glucose were constructed based on GWAS among Europeans for fasting insulin (*N* = 151,013) and fasting glucose (*N* = 200,622), respectively [37]. Among all clumping and thresholding combinations, we selected the PRS with the highest Pearson’s correlation with child HOMA-IR and fasting glucose (*N* = 894, measured at age ~8 years old).

Here, we investigated the intelligence quotient (IQ) scores assessed based on the Wechsler Intelligence Scale for Children 3^rd^ Ed (WISC-III, *N* = 6371) administered at age ~8.5 years given that WASI-II and WIAT-III (i.e., cognitive assessments used in the main analysis) were not administered during the childhood follow-ups of the ALSPAC cohort. Specifically, we investigated the associations of child and maternal PRS with age-scaled verbal IQ and performance IQ. We adjusted for child sex, highest maternal educational level attained, household income per week, gestational age of the child at birth, maternal BMI (estimated based on height and weight measured at the 12^th^ weeks of gestation), and maternal diabetes status (existing diabetes or GDM). For the associations of child PRS, we additionally adjusted for maternal PRS for the corresponding glycaemic trait. We also performed a mediation analysis focusing on the potential mediating effects of GDM, gestational age of the child at birth, birth weight, duration of breastfeeding, childhood BMI, and maternal mental health assessed at gestational week 18 using the Edinburgh Postnatal Depression Scale (EPDS). We applied the same regression-based counterfactual effect decomposition method as in the main analysis. Please note that the study website contains details of all the data that is available through a fully searchable data dictionary and variable search tool (http://www.bristol.ac.uk/alspac/researchers/our-data/) [38].

### Software

PRS and genetic PCs were constructed using Plink 1.9 (www.cog-genomics.org/plink/1.9/) [39]. The main association analyses, instrument variable analyses using 2SLS estimation (*ivreg* package), and 4-way decomposition mediation analyses (*regmedint* package) were performed using R 4.1.3. Investigation of gene overlap between PRS and child BMI was performed using *bedtools*.

## Results

### Descriptive analysis

Among the 1095 GUSTO participants, cognitive assessment (WASI-II at age ~7 years or WIAT-III at age ~9 years) was available among 530 children. The sub-sample with cognitive assessments was not different from the main GUSTO cohort regarding demographic factors (e.g., maternal ethnicity, highest education level attained, monthly household income), pregnancy-related factors (e.g., parity, pre-pregnancy BMI, maternal status of GDM), and child HOMA-IR and fasting glucose concentration (Table 1). However, the sub-sample was more likely to have genotyped data. Participants with child, maternal, or paternal genotyped data were not different from the full cohort (Supplementary Table 1).

**Table 1:** Cohort characteristics (GUSTO).

Variable	Category	GUSTO cohort (*N* = 1095)				Sub-sample with neurodevelopment measures (*N* = 530)^a^				*P*-value^b^
		*N*	%	Mean	SD	*N*	%	Mean	SD	
Availability of child genotype	No	70	6.4%			18	3.4%			0.017
	Yes	1025	93.6%			512	96.6%			
Availability of maternal genotype	No	70	6.4%			18	3.4%			0.017
	Yes	1025	93.6%			512	96.6%			
Availability of paternal genotype	No	406	37.1%			123	23.2%			3.1E−08
	Yes	689	62.9%			407	76.8%			
Child sex	Girls	523	47.8%			248	46.8%			0.753
	Boys	572	52.2%			282	53.2%			
Maternal ethnicity	Chinese	598	54.6%			298	56.2%			0.101
	Indian	205	18.7%			77	14.5%			
	Malay	291	26.6%			154	29.1%			
Highest maternal education level attained	No university degree	726	66.3%			367	69.2%			0.294
	With university degree	355	32.4%			158	29.8%			
Household income (per month)	S$0 to S$1999	163	14.9%			88	16.6%			0.620
	S$2000 to S$3999	324	29.6%			167	31.5%			
	S$4000 to S$5999	255	23.3%			121	22.8%			
	More than S$6000	282	25.8%			125	23.6%			
Maternal age at delivery		1095		30.9	5.1	530		30.9	5.1	0.981
Gestational age of child at birth (weeks)		1095		38.7	1.6	530		38.8	1.5	0.674
Maternal pre-pregnancy BMI (kg/m^2^)		993		22.7	4.4	482		22.7	4.5	0.941
Parity	Nulliparous	468	42.7%			225	42.5%			0.955
	Parous	627	57.3%			305	57.5%			
Maternal status of GDM	No	854	78.0%			423	79.8%			0.572
	Yes	188	17.2%			85	16.0%			
Childhood HOMA-IR		610		1.3	1.5	419		1.3	1.1	0.682
Childhood fasting glucose (mmol/L)		654		4.6	0.4	440		4.6	0.4	0.846

*BMI* body-mass index, *GDM* gestational diabetes mellitus, *HOMA-IR* homoeostatic model assessment for insulin resistance, *SD* standard deviation.

^a^Sample with available data on at least one neurodevelopment assessment of interest.

^b^*P*-values for differences comparing the participants in the GUSTO cohort and those with available data on at least one neurodevelopment assessment were estimated from chi-squared test for categorical characteristics and from t-test for continuous characteristics.

### Correlation between PRS and child glycaemic traits

Overall, child and maternal PRS were well correlated with child HOMA-IR (Supplementary Table 2**;** child PRS: *r* = 0.120; maternal PRS: *r* = 0.105) and fasting glucose (Supplementary Table 2; child PRS: *r* = 0.233; maternal PRS: *r* = 0.131). The correlation between paternal PRS and child glycaemic traits was weaker (Supplementary Table 2; HOMA-IR: *r* = 0.048; fasting glucose: *r* = 0.079).

### Polygenic risk for glycaemic traits and cognitive function

#### Parental PRS

**Fig. 1:**
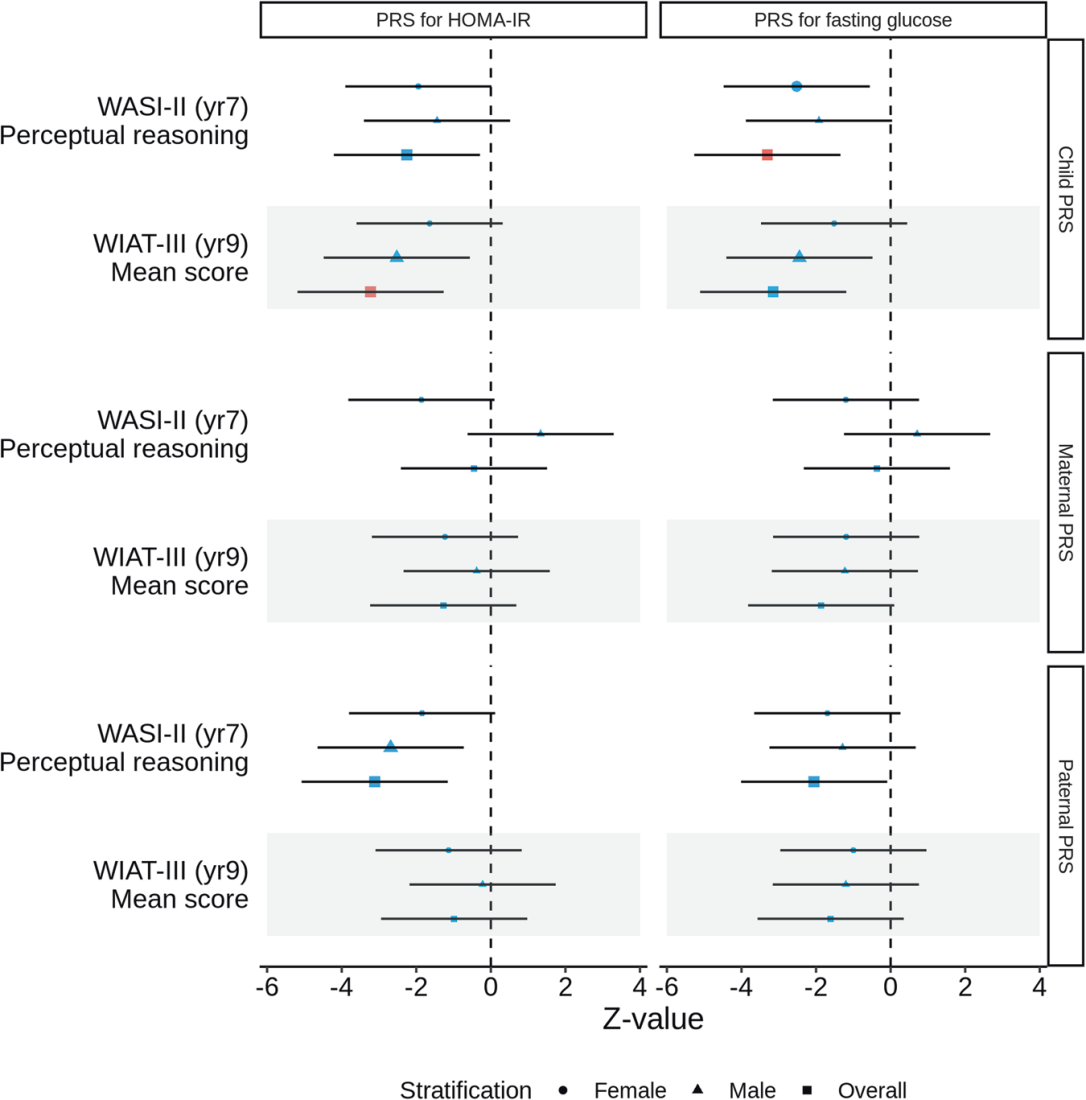
Associations of polygenic risk scores for child homoeostatic model assessment for insulin resistance (HOMA-IR) and fasting glucose with child neurodevelopment. Larger blue symbols indicate associations with a *p*-value smaller than 0.05. Red symbols indicate associations with a *p*-value smaller than 0.05/36.

We did not find associations of maternal PRS for child HOMA-IR or fasting glucose with child cognitive function under investigation; however, higher paternal PRS for HOMA-IR was suggestively associated with lower child perceptual reasoning (β = −0.172, *p*-value = 0.002, 95%CI −0.280 to −0.064; Table 2 and Fig. 1). Excluding SNPs that overlap with birth weight and child BMI genes did not have a substantial influence on these findings (Supplementary Table 3 and Supplementary Fig. 1). No mediating effects were identified for these associations (Supplementary Table 4 and Supplementary Figs. 2–5).

**Table 2:** Associations of polygenic risk scores for insulin resistance and fasting glucose with child neurodevelopment.

PRS sample	Outcome	Stratification	PRS for HOMA-IR^a^						PRS for fasting glucose^a^					
			*N*	Beta	SE	*P*-value	95% CI (lower)	95% CI (upper)	*N*	Beta	SE	*P*-value	95% CI (lower)	95% CI (upper)
Child	WASI-II (yr7) Perceptual reasoning	Female	141	−0.178	0.092	0.052	−0.358	0.002	124	−0.111	0.133	0.406	−0.372	0.151
		Male	160	−0.128	0.089	0.149	−0.301	0.046	131	−0.088	0.095	0.359	−0.275	0.099
		Overall	301	−0.141	0.063	0.024	−0.264	−0.018	255	−0.101	0.076	0.184	−0.250	0.048
	WIAT-III (yr9) Mean score	Female	114	−0.172	0.105	0.101	−0.378	0.033	102	−0.033	0.140	0.812	−0.308	0.241
		Male	121	−0.278	0.110	0.012	−0.494	−0.062	101	−0.237	0.133	0.075	−0.497	0.023
		Overall	235	−0.222	0.069	0.001*	−0.357	−0.087	203	−0.169	0.084	0.045	−0.334	−0.004
Maternal	WASI-II (yr7) Perceptual reasoning	Female	183	−0.134	0.072	0.063	−0.276	0.007	154	−0.057	0.073	0.436	−0.201	0.087
		Male	200	0.092	0.069	0.182	−0.043	0.227	162	−0.015	0.075	0.847	−0.162	0.132
		Overall	383	−0.022	0.048	0.651	−0.116	0.072	316	−0.036	0.048	0.446	−0.130	0.057
	WIAT-III (yr9) Mean score	Female	145	−0.107	0.087	0.219	−0.278	0.064	123	−0.162	0.094	0.084	−0.345	0.022
		Male	150	−0.038	0.101	0.704	−0.236	0.159	123	0.061	0.082	0.453	−0.099	0.221
		Overall	295	−0.075	0.059	0.202	−0.191	0.040	246	−0.055	0.059	0.349	−0.171	0.060
Paternal	WASI-II (yr7) Perceptual reasoning	Female	142	−0.162	0.088	0.065	−0.334	0.010	125	0.088	0.109	0.418	−0.125	0.301
		Male	167	−0.201	0.075	0.007	−0.347	−0.054	137	0.041	0.099	0.683	−0.154	0.235
		Overall	309	−0.172	0.055	0.002	−0.280	−0.064	262	0.052	0.068	0.444	−0.081	0.184
	WIAT-III (yr9) Mean score	Female	115	−0.132	0.117	0.257	−0.361	0.097	103	−0.067	0.114	0.559	−0.291	0.157
		Male	127	−0.026	0.116	0.825	−0.253	0.201	107	0.052	0.094	0.578	−0.132	0.236
		Overall	242	−0.072	0.074	0.325	−0.216	0.072	210	0.001	0.064	0.991	−0.124	0.126

*CI* confidence interval, *HOMA-IR* homoeostatic model assessment for insulin resistance, *SE* standard error.

*The asterisk symbol indicates the association had a *p*-value smaller than the multiple comparisons *p*-value threshold of 0.05/36.

^a^We adjusted for ethnicity using the first three principal components (PCs) based on child genotype, child sex, age at glycaemic trait measurement, age at neurodevelopmental outcome assessment, highest maternal education level attained, household income, maternal age at delivery, parity, maternal pre-pregnancy body mass index (BMI), gestational age of child at birth, and maternal status of gestational diabetes mellitus (GDM). For the associations between child PRS and neurodevelopmental outcomes, we additionally adjusted for maternal and paternal PRS based on child PRS SNPs to account for the effect of parental genomes.

#### Child PRS

Higher child PRS for HOMA-IR was associated with lower WASI-II perceptual reasoning score and WIAT-III mean score (Table 2 and Fig. 1). The inverse associations of child PRS for HOMA-IR with WIAT-III mean score (β = −0.222, *p*-value = 0.001, 95% CI −0.357 to −0.087) remained significant after accounting for multiple comparisons. This association were consistent in direction among boys and girls. Higher child PRS for fasting glucose was suggestively associated with a lower WIAT-III mean score (Table 2 and Fig. 1; β = −0.169, *p*-value = 0.045, 95% CI −0.334 to −0.004).

Associations of PRS for child HOMA-IR excluding SNPs that overlap with birth weight and child BMI genes with cognitive function were consistent with the main analysis (Supplementary Table 3 and Supplementary Fig. 1). The associations in boys were more evident (WASI-II perceptual reasoning: β = −0.250, *p*-value = 0.007, 95% CI −0.433 to −0.068; WIAT-III mean score: β = −0.346, *p*-value = 0.005, 95% CI −0.587 to −0.104).

**Fig. 2:**
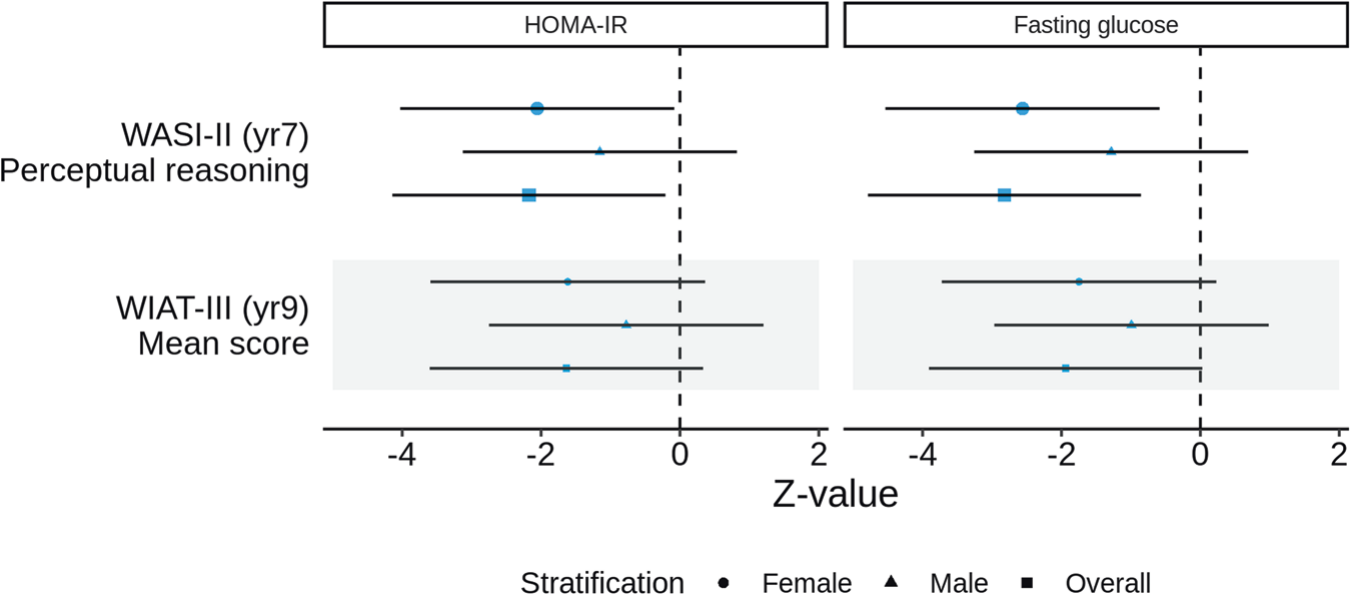
Associations of mid-childhood child homoeostatic model assessment for insulin resistance (HOMA-IR) and fasting glucose with neurodevelopment using instrumental variable regression with child polygenic risk score as a genetic instrument. Larger blue symbols indicate associations with a *p* value <0.05.

Genetically predicted HOMA-IR were nominally associated with a lower perceptual reasoning score (Table 3 and Fig. 2; β = −1.20, *p*-value = 0.030, 95% CI −2.29 to −0.12). The associations were more evident in girls than in boys. We did not find mediating effects via the child or parental phenotypes under investigation (Supplementary Table 4 and Supplementary Figs. 6 and 7).

**Table 3:** Associations of mid-childhood HOMA-IR and fasting glucose with neurodevelopment using instrumental variable regression.

Exposure	Outcome	Stratification	Sample size	Beta^a^	SE	*P*-value	95% CI (lower)	95% CI (upper)
HOMA-IR	WASI-II (yr7) Perceptual reasoning	Female	176	−1.29	0.63	0.041	−2.53	−0.05
		Male	191	−1.16	1.00	0.250	−3.14	0.82
		Overall	367	−1.20	0.55	0.030	−2.29	−0.12
	WIAT-III (yr9) Mean score	Female	141	−0.54	0.33	0.109	−1.20	0.12
		Male	151	−0.71	0.91	0.441	−2.51	1.10
		Overall	292	−0.58	0.35	0.103	−1.27	0.12
Fasting glucose	WASI-II (yr7) Perceptual reasoning	Female	180	−0.29	0.26	0.271	−0.80	0.23
		Male	203	0.43	0.43	0.328	−0.43	1.28
		Overall	383	0.02	0.21	0.921	−0.39	0.43
	WIAT-III (yr9) Mean score	Female	145	−0.27	0.28	0.341	−0.82	0.28
		Male	156	−0.13	0.30	0.661	−0.72	0.46
		Overall	301	−0.20	0.20	0.315	−0.58	0.19

*CI* confidence interval, *HOMA-IR* homoeostatic model assessment for insulin resistance, *SE* standard error.

^a^We adjusted for ethnicity using the first three principal components (PCs) based on child genotype, child sex, age at glycaemic trait measurement, and age at neurodevelopmental outcome assessment.

### Replication analysis using the ALSPAC birth cohort

Characteristics of the ALSPAC cohort are shown in Supplementary Table 5. Compared to the full ALSPAC cohort (unrelated White Caucasian participants), the mothers from the sub-samples with WISC-III assessment, child genotype, or maternal genotype were more likely to have a university degree and higher family income per week. Correlations of child and maternal PRS with the corresponding childhood glycaemic trait ranged from 0.088 to 0.150 (Supplementary Table 6), which were lower than those in GUSTO. Maternal PRS for HOMA-IR was inversely associated with WISC-III performance IQ score in girls (β = −0.045, *p*-value = 0.032, 95% CI −0.086 to −0.004; Supplementary Table 7 and Supplementary Fig. 8), but not among boys. On the other hand, child PRS for fasting glucose was inversely associated with WISC-III verbal IQ score among boys (β = −0.068, *p*-value = 0.017, 95% CI −0.125 to −0.012). We did not find mediating pathways via the potential mediators under investigation (Supplementary Table 8, Supplementary Figs. 9 and 10).

## Discussion

Studies have shown that dysfunctional insulin signalling can worsen pathology related to Alzheimer’s disease, suggesting insulin resistance is a link between metabolic syndrome and neurodegenerative disorders [40]. The relationships of early-life insulin resistance or fasting glucose concentration with cognitive function are less studied. To our knowledge, this is the first study to explore the relationships between genetic risk for child glycaemic dysregulation and early-life cognitive function. We constructed PRS for child glycaemic traits using child, maternal, and paternal genotypes in an Asian parent-offspring cohort (GUSTO) and found that child PRS and parental PRS were differently associated with cognitive function. Most consistently, we found that a higher child polygenic risk for HOMA-IR was associated with lower scores for perceptual reasoning (WASI-II) and academic performance (WIAT-III mean score). These associations were not explained by parental genotype nor measured behavioural mediators and were likely mediated via the corresponding glycaemic trait. A higher paternal polygenic risk for child HOMA-IR was also found to be associated with a lower score for perceptual reasoning in the GUSTO cohort. The impacts of PRS for HOMA-IR on cognitive function may be independent of birth weight and child BMI since the exclusion of relevant SNPs did not substantially affect the findings.

By using instrumental variable analysis, we also showed that genetically predicted glycaemic traits were associated with perceptual reasoning, suggesting a biological mechanism via glycaemic traits such as insulin resistance. These findings were more evident in girls, which may suggest sex-specific mechanisms underlying the main association between genetic risk for child glycaemic dysregulation and cognitive function. Further investigation on the sex-specific associations may provide insight into the underlying mechanisms of our findings. On the other hand, the observed associations may also be attributed to gene-environment correlation. Children’s genotypes inherited from their parents may be correlated with the environment in which they are raised [41]. This mechanism may not directly involve altering insulin resistance status or fasting glucose concentration; instead, behavioural and environmental factors such as diet and physical activity, could be relevant. With the existing data in our cohort, we did not find any mediating effects via such potential mediating factors. However, uninterrogated pathways may exist and future investigation on other mechanisms is a critical gap. Both maternal and paternal factors have been associated with offspring health, including neurodevelopment [42, 43]. Although the offspring genotype is inherited equally from both parents, the genetic contributions from the paternal genotype and the maternal genotype are not equal [44]. Experimental evidence has shown that paternal and maternal genomes contribute differently to the brain structure [45]. This may explain the differential associations of paternal and maternal PRS in our GUSTO analysis. However, replication analysis in a European birth cohort (ALSPAC) showed that higher maternal PRS for HOMA-IR was associated with a lower performance IQ score in girls. This may suggest different roles of parental genomes and that the relationship between polygenic risk for glycaemic regulation and neurodevelopment may be specific to neurodevelopmental domains and populations. Such differences in different populations suggest further research is warranted on whether these associations are robust to different environments.

In this study, we demonstrated the relevance of PRS from East Asian GWAS in a multi-ethnic Asian cohort, an understudied population with potentially higher metabolic susceptibility. The family-based design of the GUSTO cohort provided an opportunity to investigate the effects of child and parental genomes. Our replication analysis in the ALSPAC birth cohort suggests potentially ethnicity-specific associations. This encourages further enhancement of population diversity in genomic studies to advance understanding of differential genetic risk in less represented populations in the present literature. Nevertheless, limitations exist. First, GWAS of type 2 diabetes or glycaemic traits in Asian children and adolescents were not available. Thus, we chose to construct PRS for the GUSTO cohort using summary statistics from the largest GWAS among East Asian adults, which may not be the most relevant age group for child metabolic risk. However, our PRS were well correlated with child glycaemic traits. Second, the potential risk of overfitting in PRS construction cannot be fully avoided. However, the replication analysis in ALSPAC served as an independent target cohort and provided additional evidence for our analysis in GUSTO. Third, associations of polygenic risk for child glycaemic dysregulation with cognitive function do not elucidate the underlying mechanisms. The associations between a PRS and neurodevelopment may reflect shared genetic aetiology and gene-environment correlation. However, our instrumental variable analysis suggests at least part of the effect of the genetic burden of insulin resistance on cognitive function is exerted via the corresponding glycaemic traits. Nevertheless, further investigation of the functions of PRS SNPs may help reveal other relevant pathways. Fourth, genetic associations for birth weight and child BMI were obtained from a GWAS of European ancestry. Novel loci may be identified from GWAS of Asian ancestry with large sample sizes; however, such resources are not currently available. Fifth, the cognitive assessment under investigation in the replication analysis using the ALSPAC cohort was different from what we used for the main analysis using the GUSTO cohort. However, given its level of complexity, neurodevelopment needs to be evaluated using various assessment tools. Triangulation of evidence based on different neurodevelopmental assessments helps consolidate the findings.

In summary, our findings suggest a link between the child’s genetic burden of early-life glycaemic traits and lower cognitive scores in both Asian and European birth cohorts. These associations were not explained by parental genotype nor measured behavioural mediators. Nonetheless, differential findings for paternal and maternal genomes between the cohorts may be attributed to environmental differences, not merely genetic ancestry. Further investigation of the functions of the genetic structure and function of ancestry-specific PRS and more comprehensive investigations of behavioural mediators may help to better understand these findings. Given the complexity of neurodevelopment, children who are not clinically diagnosed with a disorder could still experience developmental challenges that may not be captured by cognitive assessment. Molecular markers such as neurology-related proteomics may be useful for evaluating the risk of developmental delay. Such molecular markers can also provide insight into biological mechanisms. Ultimately, further enhancement of population diversity in genomic studies is crucial to advancing the understanding of differential genetic risk in the less-represented population.

### Supplementary information

Supplementary Table

Supplementary Figure

## Data Availability

The code is available upon request.
