# Supplementary material for: Parental and child genetic burden of glycaemic dysregulation and early-life cognitive development: an Asian and European prospective cohort study
Source: Transl Psychiatry. 2024 Jan 4;14:2. doi: 10.1038/s41398-023-02694-x (PMC10766615; doi:10.1038/s41398-023-02694-x)
Supplement: Supplementary file 2 — Supplementary Figure [file 41398_2023_2694_MOESM2_ESM.docx]

**Parental and child genetic burden of glycaemic dysregulation and early-life cognitive development: an Asian and European prospective cohort study**

**Supplementary Figures**

Supplementary Figure 1 Associations of polygenic risk scores (PRS) for child homeostatic model assessment for insulin resistance (HOMA-IR) and fasting glucose with child neurodevelopment. PRS was constructed by excluding single nucleotide polymorphisms (SNPs) annotated to genes/transcripts associated with birthweight and child body-mass index.

Supplementary Figure 2 Mediation analysis for the associations of maternal polygenic risk scores and perceptual reasoning.

Supplementary Figure 3 Mediation analysis for the associations of maternal polygenic risk scores and WIAT-III mean score

Supplementary Figure 4 Mediation analysis for the associations of paternal polygenic risk scores and perceptual reasoning

Supplementary Figure 5 Mediation analysis for the associations of paternal polygenic risk scores and WIAT-III mean score

Supplementary Figure 6 Mediation analysis for the associations of child polygenic risk scores and perceptual reasoning

Supplementary Figure 7 Mediation analysis for the associations of child polygenic risk scores and WIAT-III mean score

Supplementary Figure 8 Associations of polygenic risk scores (PRS based on genome-wide association study for fasting glucose) for child homeostatic model assessment for insulin resistance (HOMA-IR) and fasting glucose with child neurodevelopment assessed based on the Wechsler Intelligence Scale for Children 3rd Ed (WISC-III, age ~8.5 years old) (ALSPAC cohort)

Supplementary Figure 9 Mediation analysis for the associations of child polygenic risk scores and WISC-III verbal IQ score in boys (ALSPAC cohort)

Supplementary Figure 10 Mediation analysis for the associations of maternal polygenic risk scores and WISC-III performance IQ score among girls (ALSPAC cohort)


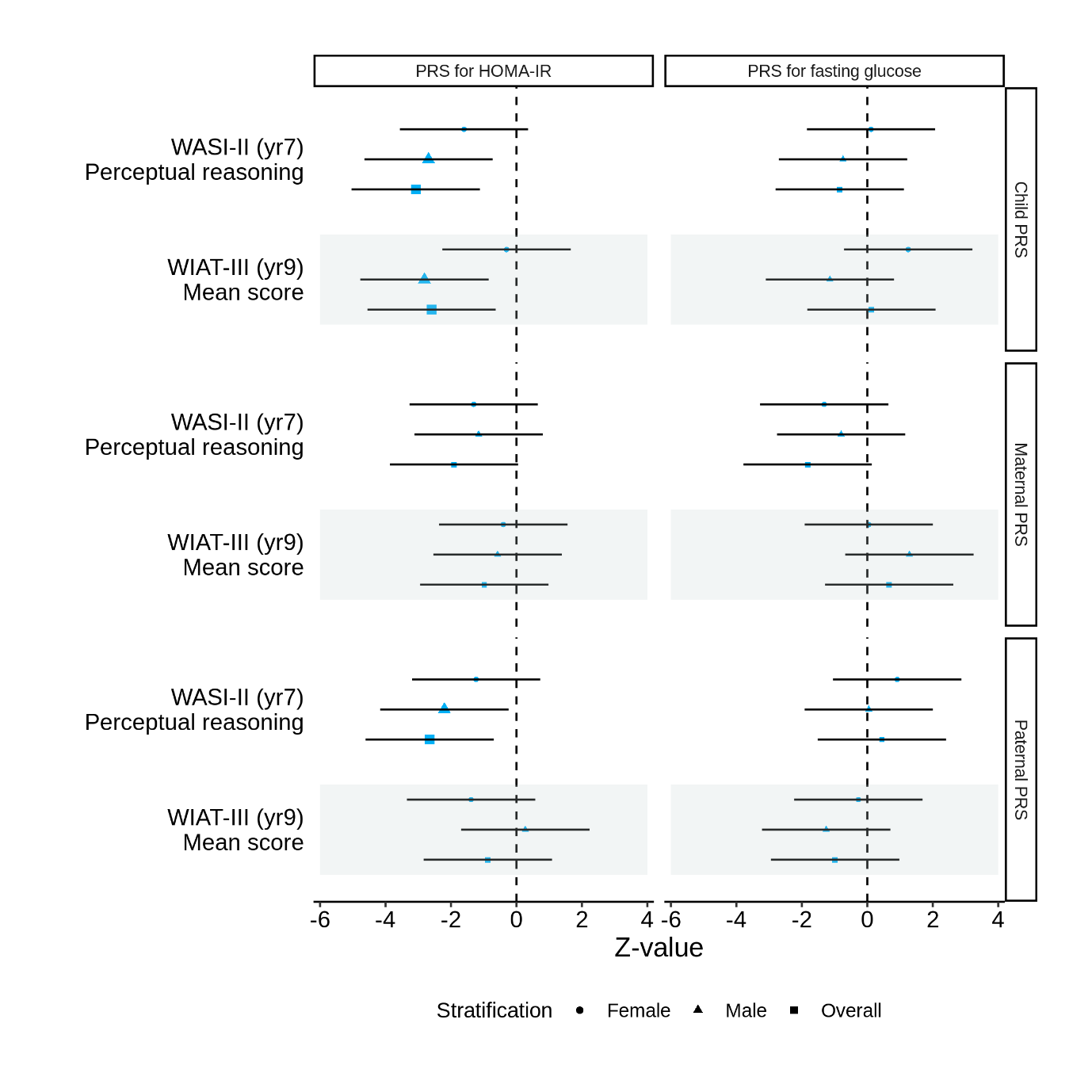


Figure 1 Associations of polygenic risk scores (PRS) for child homeostatic model assessment for insulin resistance (HOMA-IR) and fasting glucose with child neurodevelopment. PRS was constructed by excluding single nucleotide polymorphisms (SNPs) annotated to genes/transcripts associated with birthweight and child body-mass index. Larger blue symbols indicate associations with a p-value smaller than 0.05.


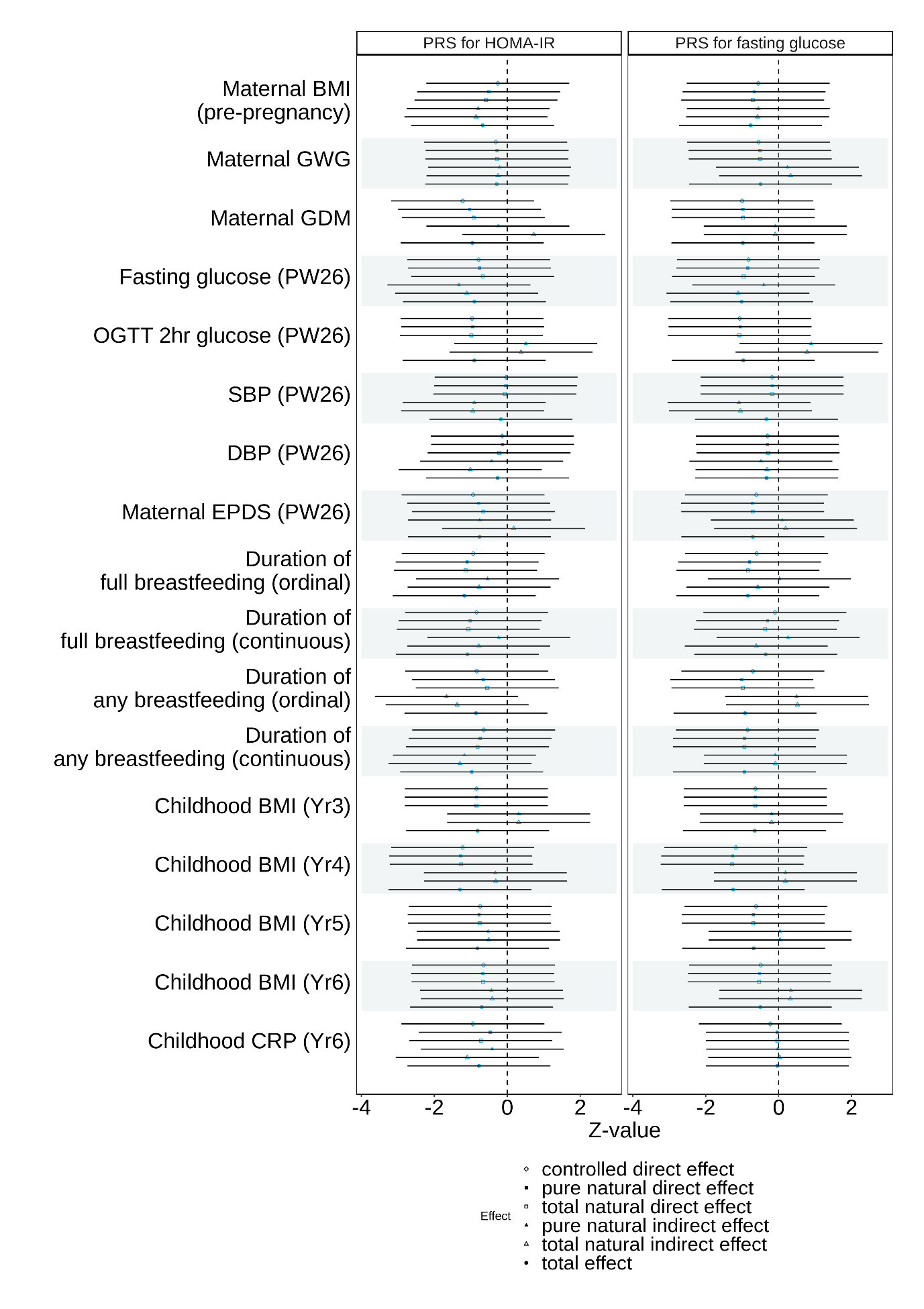


Figure 2 Mediation analysis for the associations of maternal polygenic risk scores and perceptual reasoning (75^th^ quantile vs 25^th^ quantile of the polygenic risk scores (PRS)). Red symbols indicate effects with P-value smaller than 0.05. (GWG: gestational weight growth; GDM: gestational diabetes; OGTT: oral glucose tolerance test; PW: pregnancy week; SBP: systolic blood pressure; DBP: diastolic blood pressure; BMI: body-mass index; CRP: C-reactive protein; EPDS: Edinburgh Postnatal Depression Scale)


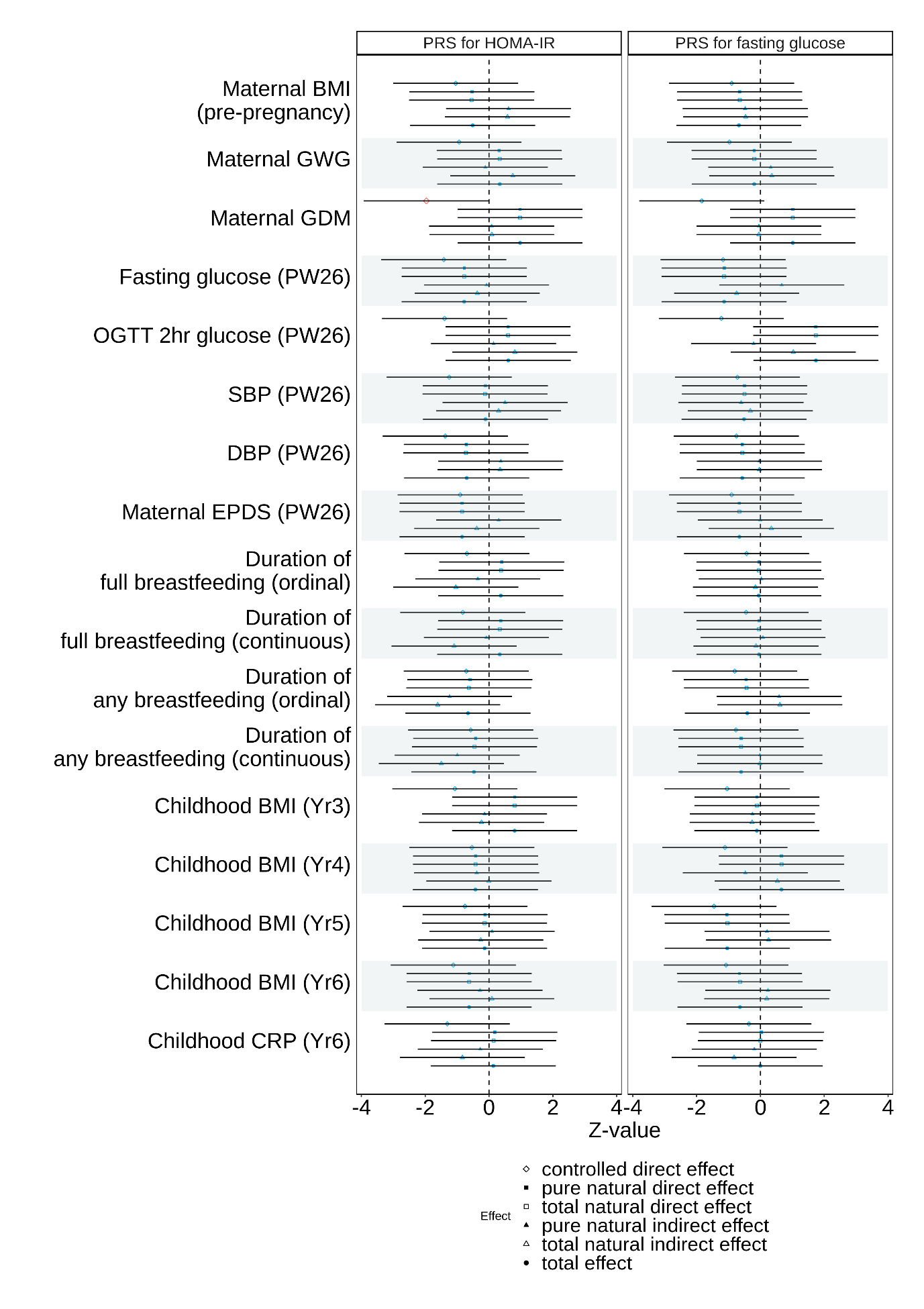


Figure 3 Mediation analysis for the associations of maternal polygenic risk scores and WIAT-III mean score (75^th^ quantile vs 25^th^ quantile of the polygenic risk scores (PRS)). Red symbols indicate effects with P-value smaller than 0.05. (GWG: gestational weight growth; GDM: gestational diabetes; OGTT: oral glucose tolerance test; PW: pregnancy week; SBP: systolic blood pressure; DBP: diastolic blood pressure; BMI: body-mass index; CRP: C-reactive protein; EPDS: Edinburgh Postnatal Depression Scale)


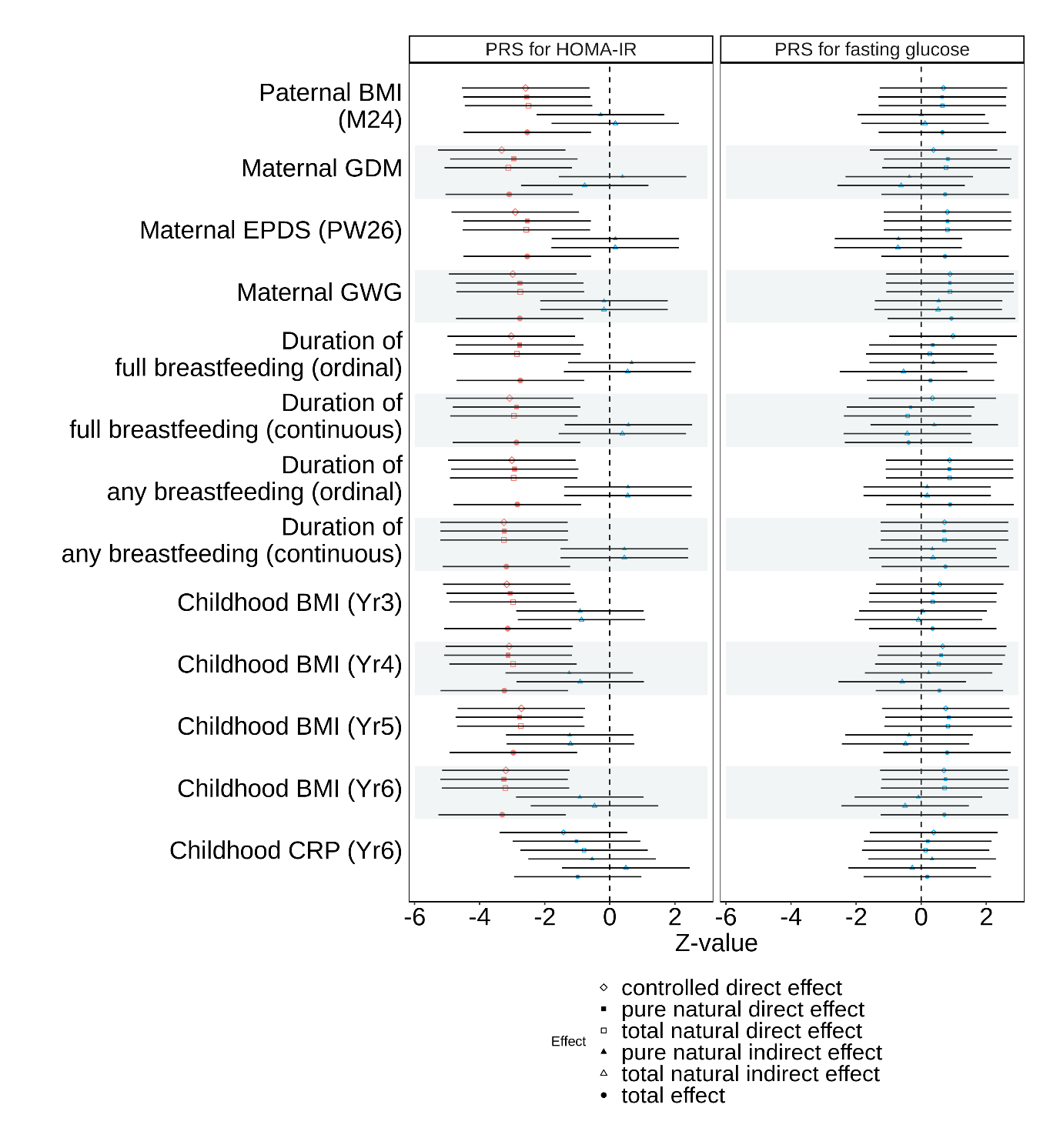


Figure 4 Mediation analysis for the associations of paternal polygenic risk scores and perceptual reasoning (75^th^ quantile vs 25^th^ quantile of the polygenic risk scores (PRS)). Red symbols indicate effects with P-value smaller than 0.05. (GWG: gestational weight growth; GDM: gestational diabetes; OGTT: oral glucose tolerance test; PW: pregnancy week; SBP: systolic blood pressure; DBP: diastolic blood pressure; BMI: body-mass index; CRP: C-reactive protein; EPDS: Edinburgh Postnatal Depression Scale)


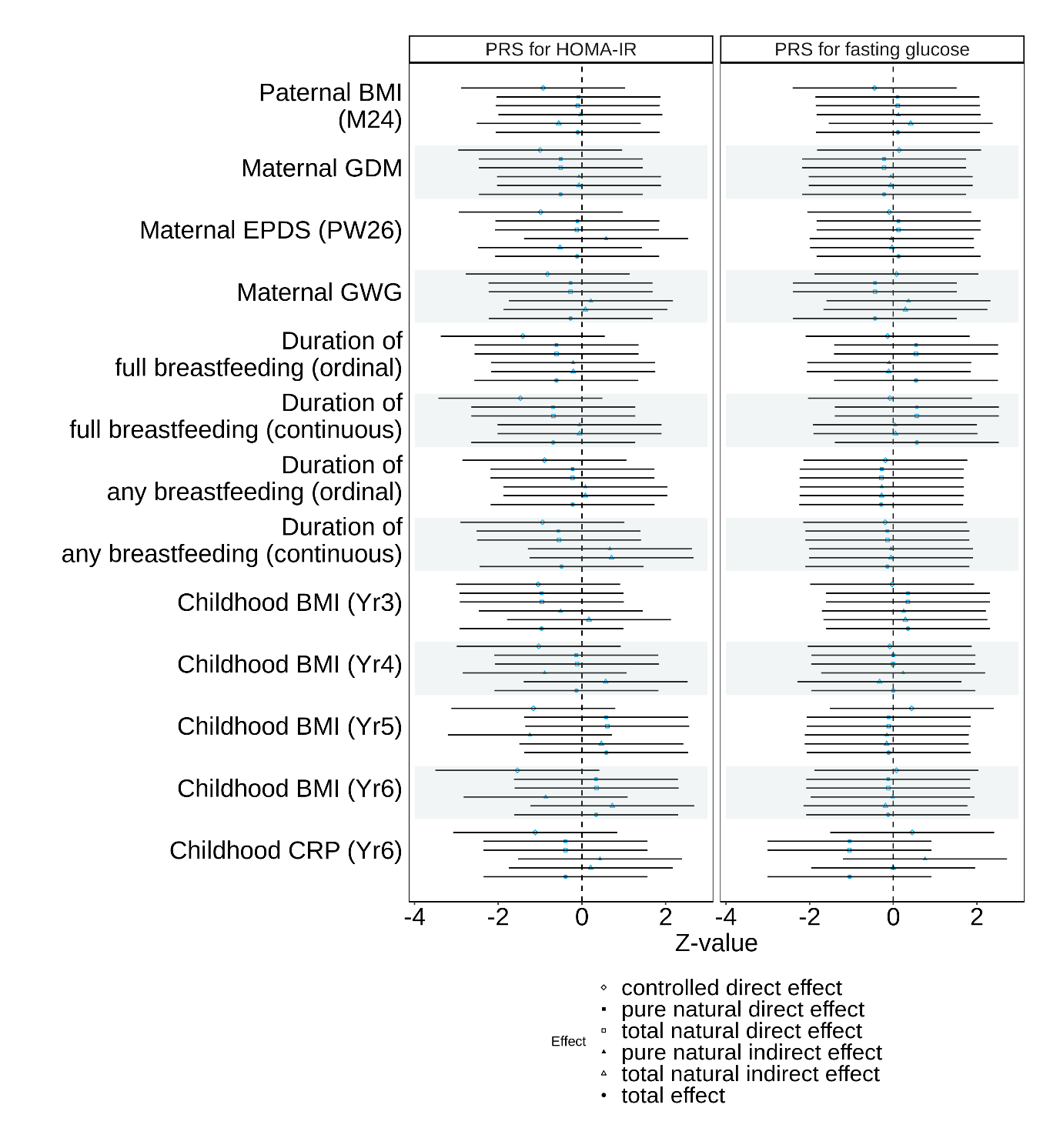


Figure 5 Mediation analysis for the associations of paternal polygenic risk scores and WIAT-III mean score (75^th^ quantile vs 25^th^ quantile of the polygenic risk scores (PRS)). Red symbols indicate effects with P-value smaller than 0.05. (GWG: gestational weight growth; GDM: gestational diabetes; OGTT: oral glucose tolerance test; PW: pregnancy week; SBP: systolic blood pressure; DBP: diastolic blood pressure; BMI: body-mass index; CRP: C-reactive protein; EPDS: Edinburgh Postnatal Depression Scale)


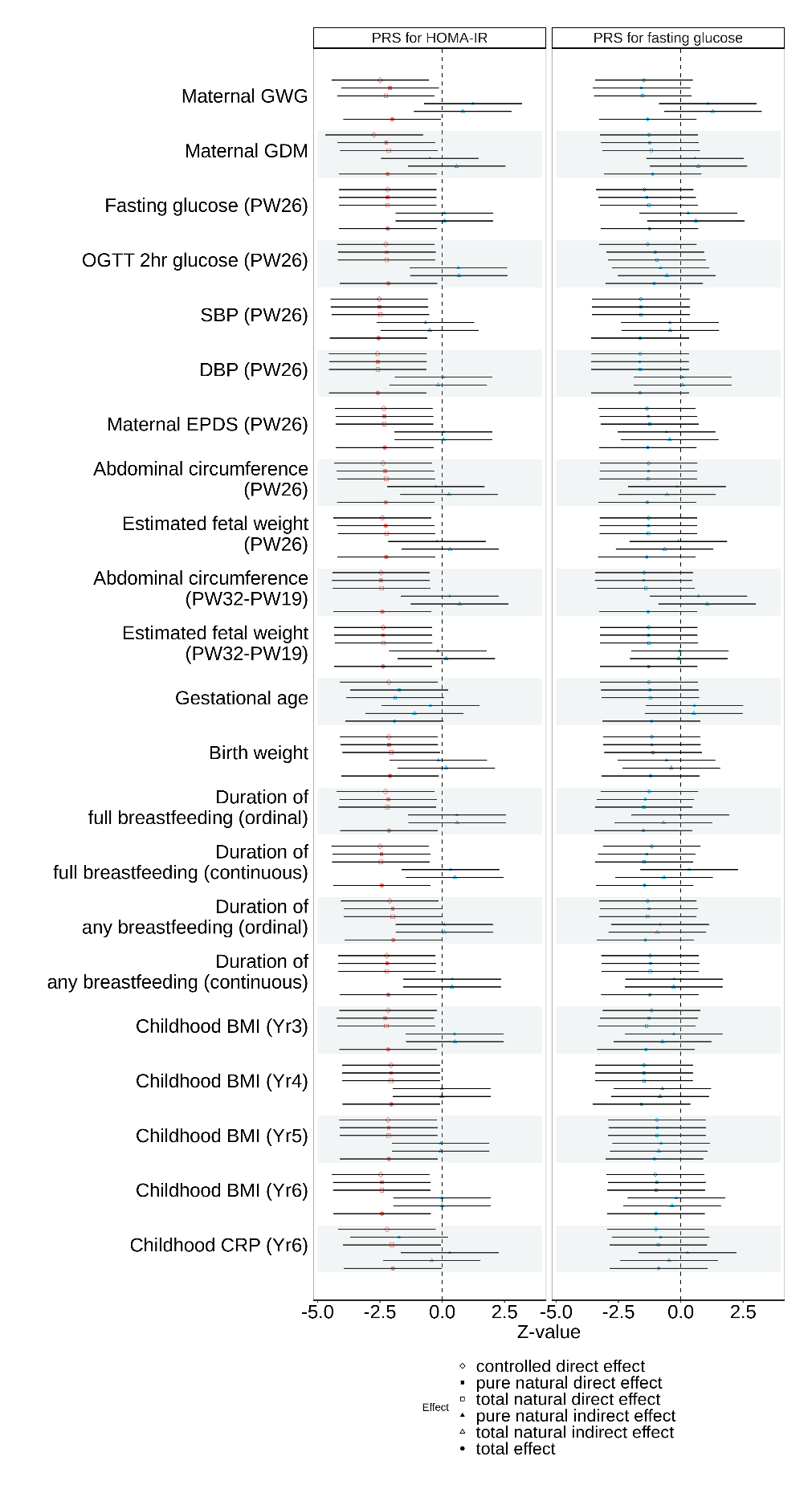


Figure 6 Mediation analysis for the associations of child polygenic risk scores and perceptual reasoning (75^th^ quantile vs 25^th^ quantile of the polygenic risk scores (PRS)). Red symbols indicate effects with P-value smaller than 0.05. (GWG: gestational weight growth; GDM: gestational diabetes; OGTT: oral glucose tolerance test; PW: pregnancy week; SBP: systolic blood pressure; DBP: diastolic blood pressure; BMI: body-mass index; CRP: C-reactive protein; EPDS: Edinburgh Postnatal Depression Scale)


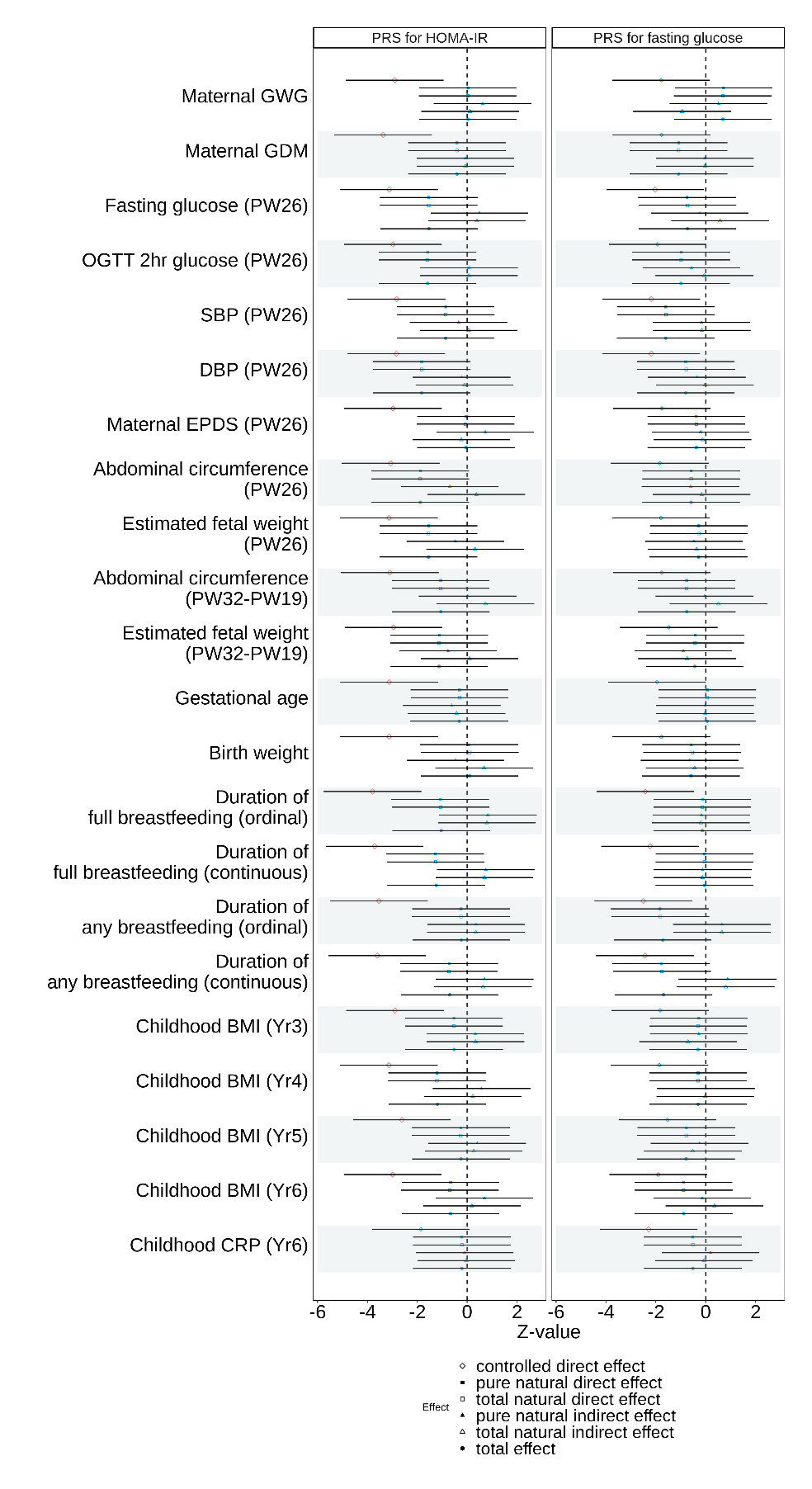


Figure 7 Mediation analysis for the associations of child polygenic risk scores and WIAT-III mean score (75^th^ quantile vs 25^th^ quantile of the polygenic risk scores (PRS)). Red symbols indicate effects with P-value smaller than 0.05. (GWG: gestational weight growth; GDM: gestational diabetes; OGTT: oral glucose tolerance test; PW: pregnancy week; SBP: systolic blood pressure; DBP: diastolic blood pressure; BMI: body-mass index; CRP: C-reactive protein; EPDS: Edinburgh Postnatal Depression Scale)


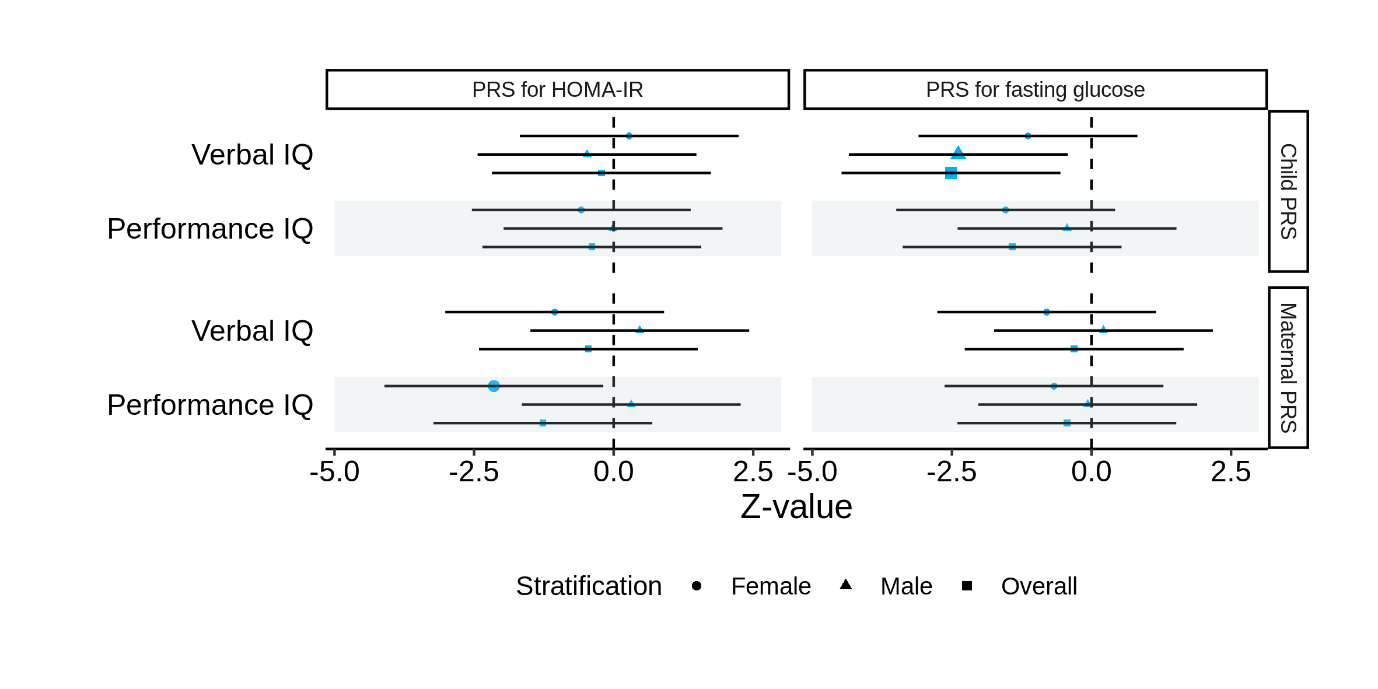


Figure 8 Associations of polygenic risk scores for child homeostatic model assessment for insulin resistance (HOMA-IR) and fasting glucose with child neurodevelopment assessed based on the Wechsler Intelligence Scale for Children 3rd Ed (WISC-III, age ~8.5 years old) (ALSPAC cohort). Larger blue symbols indicate associations with a p-value smaller than 0.05.


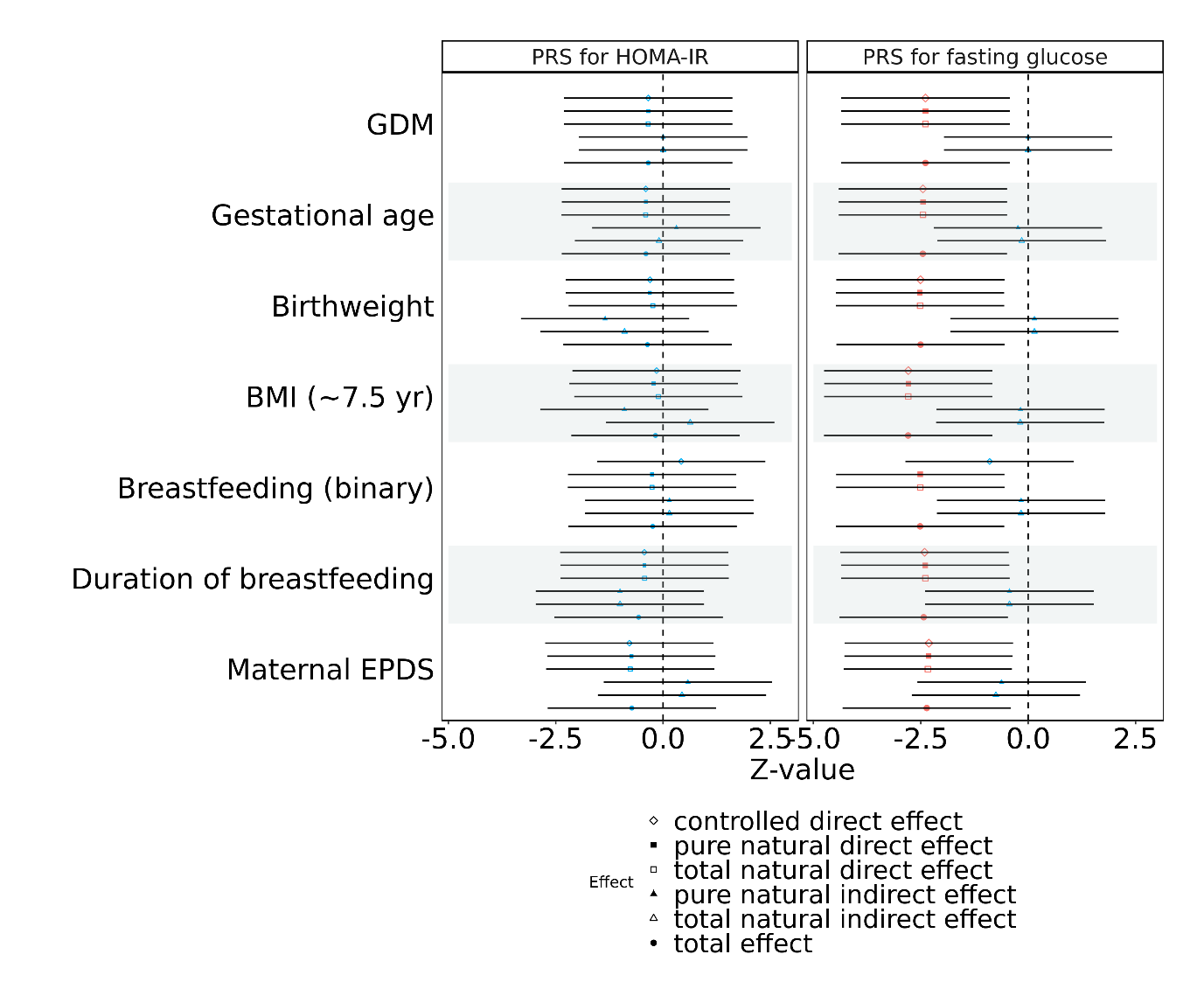


Figure 9 Mediation analysis for the associations of child polygenic risk scores and WISC-III verbal IQ score in boys (ALSPAC cohort; 75th quantile vs 25th quantile of the polygenic risk scores (PRS)). Red symbols indicate effects with P-value smaller than 0.05. This model adjusted for maternal educational level, household income, gestational age, maternal BMI, and maternal diabetes status (GDM: gestational diabetes; BMI: body-mass index; EPDS: Edinburgh Postnatal Depression Scale).


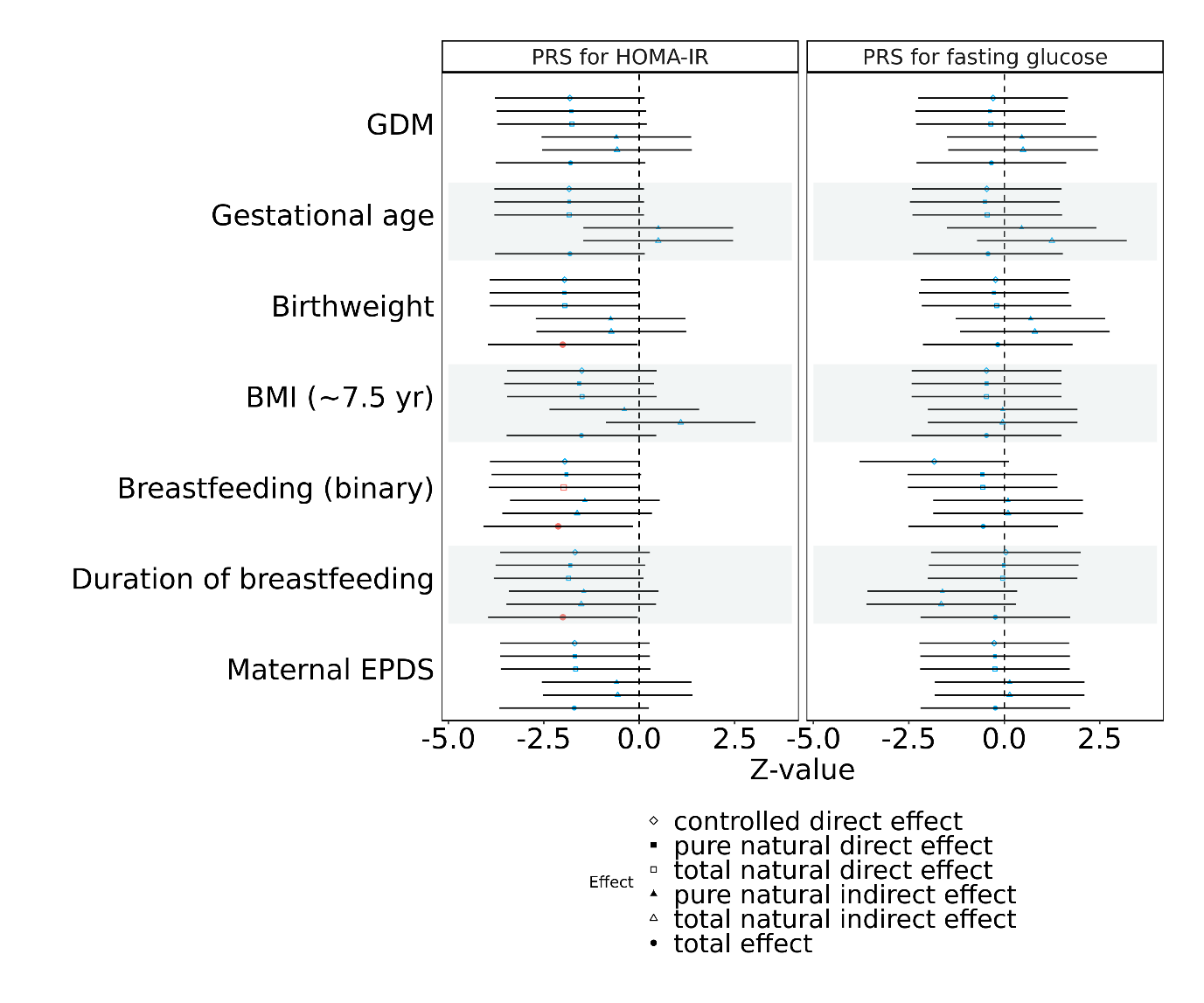


Figure 10 Mediation analysis for the associations of maternal polygenic risk scores and WISC-III performance IQ score among girls (ALSPAC cohort ; 75th quantile vs 25th quantile of the polygenic risk scores (PRS)). Red symbols indicate effects with P-value smaller than 0.05. This model adjusted for maternal educational level and household income (GDM: gestational diabetes; BMI: body-mass index; EPDS: Edinburgh Postnatal Depression Scale)
